# Establishing community mental health facilities: a comparative review of Hong Kong and international jurisdictions

**DOI:** 10.1186/s12913-022-08868-5

**Published:** 2023-01-07

**Authors:** Xiaoting Ou, Vincent W. P. Lee, Daniel W. L. Lai

**Affiliations:** 1 Department of Sport, Physical Education and Health, Hong Kong Baptist University, Kowloon Tong, Hong Kong; 2 Department of Social Work, Hong Kong Baptist University, Kowloon Tong, Hong Kong; 3 Faculty of Social Sciences, Hong Kong Baptist University, Kowloon Tong, Hong Kong

**Keywords:** Community opposition, Discrimination, Mental illness, Stigma, NIMBY

## Abstract

**Background:**

The establishment of mental health facilities in the community has been hindered by opposition from local residents in Hong Kong. Through a comparative review, this study aimed to compare the issues related to the process of establishment of community-based mental health facilities between Hong Kong and selected overseas countries and regions. It will better inform the strategies and best practices that can be adopted for the establishment of mental health facilities in Hong Kong.

**Methods:**

Three electronic databases (PubMed, Scopus, and PsycINFO) were used to examine literature on nine jurisdictions in Asia and western societies from 2005 to 2019. In addition, we conducted a number of in-depth interviews with overseas experts to gain in-depth insights and clarify information that was unavailable or unclear. A total of 19,248 articles were identified through the initial search. 71 of them met the inclusion criteria. In addition, 20 articles about the establishment of other types of community facilities or sensitive facilities were identified from supplementary sources.

**Results:**

Most Western countries and Singapore have adopted regulations or laws to reduce public discrimination against particular groups, giving them corresponding human rights and legislating to demarcate the use of land in the community. Regions close to Hong Kong emphasize communication with community leaders to obtain support for sensitive services or facilities.

**Conclusions:**

Hong Kong may consider strengthening the land zoning ordinance in relation to community sensitive facilities, as well as increasing communication with the community and considering the possibility of locating facilities in government buildings.

## Background

Over the past 20 years, the global number of disability-adjusted life years (DALYs) due to mental illness has increased nearly 155-fold and the rate has increased by nearly 2%, especially in high-income countries [1]. A study conducted by the World Health Organization (WHO) found that about one in eight people have a mental illness [2].

In Hong Kong, one in seven people live with a mental illness. The number of people with mental illness has been doubled in just 10 years. However, more than 70% of people with mental illness do not seek any professional help [3]. Given the inadequacy of mental health services, the Hong Kong Government announced in its 2009–2010 Policy Address the restructuring of community mental health support services through the establishment of Integrated Community Centers for Mental Wellness (ICCMWs). However, the site selection of ICCMWs has been difficult due to numerous oppositions from local communities. The Equal Opportunities Commission (EOC) [4] found that in the public consultation process concerning the siting of the ICCMWs, at least nine of them encountered community opposition. Other than feeling unsafe with the influx of service users whom they perceived as different from themselves, residents also perceived persons with mental illness as violent and causes of disturbance and crime [5]. These stereotypical attitudes in Hong Kong have not changed in past decades [6].

Stigmatization and discrimination against the establishment of services for people with mental illness are common in different jurisdictions [7, 8]. Structural discrimination is reflected through negative attitudes, such as opposition to allocation of financial resources to the care of people with mental health and opposition to service establishment in residential communities [9]. All these negatively affect services, rehabilitation processes, and self-image of people with mental illness [9], directly and indirectly influencing their treatment seeking attitudes [10], and trust toward services [11]. Moreover, these negative effects, especially those associated with stigma attached to mental illness, also affect the implementation and enforcement of mental health policies [12, 13]. To reduce the negative effects, communities and public policies play an essential role in the rehabilitation process of people with mental illness [14]. The 2003 New Freedom Commission document on mental health in the United States identified recovery as a core principle of federal mental health policy. Recovery is defined as the process by which people with mental illness are able to live, work, and participate fully in the community [14]. Health policies profoundly impact mental health as they aim to help people with mental illness to recover. For example, the expansion of health insurance through the Patient Protection and Affordable Care Act and the Mental Health Parity and Addiction Equity Act has reduced the burden of medical care on patients, thereby improving their psychological distress [15]. In Hong Kong, the Mental Health Ordinance (Cap 136), which is relevant to the care and treatment of people with mental illness, ensures the rights of them. In addition, the Disability Discrimination Ordinance (DDO) prevents discrimination against persons with mental illness or people in recovery and their carers based on the patient's disability. In general, the community has an important influence on the rehabilitation of people with mental illness while the mental health related policies and ordinance could ensure that their rights are valued and protected from discrimination during recovery.

The ‘Not in My Backyard’ (NIMBY) phenomenon is a common challenge to the establishment of new facilities in neighborhoods. It may be related to both a lack of public knowledge and understanding of mental illness and to approaches to public consultation and engagement [16]. It is important to understand how both challenges and responses to these issues play out in different countries and contexts. However, systematic studies of policies and approaches to the establishment of community sensitive facilities are scant. This study examines existing research to understand relevant issues related to the establishment of community-based mental health facilities in Hong Kong and selected overseas countries and regions, examining how governments in different jurisdictions consider community voices and approach the processes of establishing mental health facilities.

## Methods

We adopted a systematic comparative review strategy, which serves to clarify the working definitions, key concepts, and conceptual boundaries of a topic or field and to map the main sources and available evidence [17, 18]. By comparing the differences and similarities in the empirical and theoretical focus of multiple groups of information sources, it is possible to identify meaningful factors in the different literatures or documents that can be used to provide feasible recommendations [19]. This strategy is particularly useful when a body of literature is complex and diverse or has not yet been comprehensively reviewed. No registration with PROSPERO was made since it does not accept registration of scoping review study. However, the preferred reporting items for systematic reviews and meta-analyses extension for scoping reviews (PRISMA-ScR) guideline was followed in this study [20]. Our review protocol was guided by four questions:

1. What are the issues and challenges encountered by government officials, service providers, communities, and service users related to the siting of mental health facilities?
2. What are the practices and norms adopted by governments in Hong Kong and different jurisdictions to establish mental health service facilities?
3. What are the considerations and practices involved?
4. What are the factors related to successes and failures in resolving challenges to these planning, consultation, and establishment processes?

### Search and review strategy

The literature search and review were conducted in 2019, involving keyword searches within three electronic databases: PubMed, Scopus, and PsycINFO. They were based on four dimensions of interest: mental health and mental health patients, mental health facilities, neighborhood or community responses, and policy responses (see Table 1). In order to study this topic more comprehensively, we searched grey literature in addition to the literature from the database mentioned above. We also reviewed government policies (e.g. mental health and service planning policies) and documents, records of government meetings, research reports from community organizations, and media reports. We examined literature on nine other jurisdictions in Asian and Western societies – Macau, Taiwan, Singapore, Japan, Korea, Australia, New Zealand, Canada, and the United States – so that comparison could be made. Such selection is mainly based on their geographical proximity to Hong Kong, cultural characteristics (for example, Chinese and Asian cultural norms), the mode of providing social services (including the roles of government and service providers), and legal and administrative systems. Materials about the siting of mental health facilities in communities and neighbourhoods in other jurisdictions were reviewed to assess similarities and differences between international cases, norms, and practices. Mainland China is not covered in this study because of its drastic differences as compared with the case of Hong Kong regarding the legal and administrative systems and the roles of the government and non-government organizations in offering social services. Meanwhile, Australia, New Zealand, Canada, and the United States have similar structures for operating community social services and legal systems as Hong Kong, such as public deliberation, town planning, and litigation processes. For data verification, we also consulted a number of overseas experts with scholarly and professional experience with the mental health service delivery system. The six experts were from Macao, Taiwan, Singapore, Japan, Australia, and Canada.

**Table 1:** Search terms used for scoping review

Search focus	Search string	Sources
Basic search string structure	("mental health" OR "mental disorder" OR "mental illness" OR "mental recovery" OR "disable") AND (facility OR “mental health facility” OR clinic OR “community facility”) AND (neighborhood OR community) AND ("stigma" OR "challenges" OR "discrimination" OR against) AND (Macau OR Taiwan OR Singapore OR Japan OR Korea OR Australia OR "New Zealand" OR Canada OR "United States")	In press Source type: journal Year: 2005–2019 Databases: PubMed, Scopus, and PsycINFO
Hong Kong	a. (‘legal provisions’ WITH discrimination OR discriminate OR stigma OR ‘legal regulations’ WITH discrimination OR discriminate OR stigma) AND (‘people with disabilities’ OR ‘mental illness’ OR ‘mental health’ OR’ mental health services’ OR clinic OR ‘welfare facilities’) AND (‘statutory rights’ OR ‘human right’) AND (‘community’) AND (‘Hong Kong’) b. (‘land zoning’ WITH ‘Social services’ WITH protocols OR development plan OR guidelines) AND (‘social integration’) AND (‘Hong Kong’)	Source type: legal regulations, official documents, Legislative Council minutes newspaper, online media and reports Searches were conducted of government websites and local media
Overseas countries or regions	c.(‘legal provisions’ WITH discrimination OR discriminate OR stigma OR legal regulations WITH discrimination OR discriminate OR stigma) AND (‘people with disabilities’ OR’ mental illness’ OR ‘mental health’ OR ‘mental health services’ OR clinic OR ‘welfare facilities’) AND (‘statutory rights’ OR ‘human right’) AND (community) AND (Macau OR Taiwan OR Singapore OR Japan OR Korea OR Australia OR ‘New Zealand’ OR Canada OR ‘United States’) d.(‘land zoning’ WITH ‘Social services’ WITH protocols OR development plan OR guidelines) AND (‘social integration’) AND (Macau OR Taiwan OR Singapore OR Japan OR Korea OR Australia OR New Zealand OR Canada OR ‘United States’)	Source type: legal regulations, newspaper, and reports Searches were conducted of government websites

### Inclusion and exclusion criteria

Studies included in the review were published in English peer review journals between January 2005 and June 2019. Researchers screened the titles and abstracts of the articles and included studies from social sciences, psychology, public policy and environmental science disciplines. We first screened out the literature on the establishment of mental health facilities in the nine jurisdictions covered. Literature on the establishment of other controversial community facilities in these nine countries were also retained for reference purposes. Based on the research protocol, the literature was further reviewed and grouped based on the approaches taken to establish mental health facilities in these countries. For this, different categories, such as challenges encountered, legal approaches, human rights, land policies, and negotiations, have been created to further compare the literature on the countries’ approaches to setting up mental health facilities. The systematic categorization helped to compare the approaches taken by different countries to setting up mental health facilities and to highlight the strengths of each approach for Hong Kong's reference. Only articles about the nine jurisdictions mentioned above were included, and those about the treatment or prevention of mental health problems were excluded. In addition, this literature screening approach ensures that the literature we selected was consistent with our protocol. When researchers had disagreements about the selection of literature, a discussion among researchers was conducted in order to review and consider whether the literature should be included in the study.

## Search Results

**Fig. 1:**
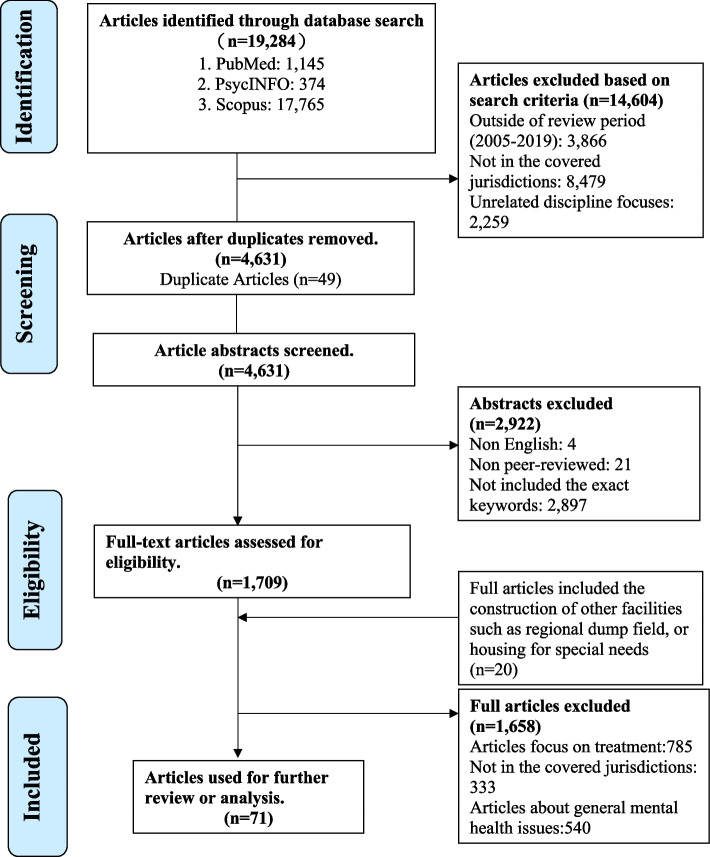
The scoping review process

A total of 19,248 articles were identified through the initial search, of which 71 met the inclusion criteria (see Fig. 1). An additional 20 articles were identified from supplementary sources. Additional articles were mainly about the establishment of other types of community facilities or sensitive facilities, such as drug rehabilitation centers, youth hostels, and accommodation centers for homeless people. Six major themes were identified as follows.

### Challenges and difficulties in establishing mental health facilities

Stigmatization and discrimination toward mental health patients are associated with misconceptions of mental illness and influenced by the mass media. Public perceptions are influenced by media reports of past tragedies involving mentally ill patients as perpetrators of violence (such as the Tsim Sha Tsui MTR Station firebomb attack in Hong Kong) [21]. Similarly, studies in the US have shown that community residents lack awareness of mental illness, have little empathy toward mental health patients, and are easily influenced by the mainstream media [22]. In New Zealand, discrimination and misunderstanding toward mental illness persist among the public, with many believing that their safety is threatened by mental health patients in their communities [23].

In Singapore, some people see mental health problems as a matter of personal weakness [24]. Lack of awareness of institutional services and fear of people with disabilities are the reasons why many people in Taiwan discourage the establishment of facilities [25]. In Macau, mental illness is treated as a rare and untreatable disease, leading to public fear and discrimination [26]. In Japan, the public perception of mental illness is relatively negative, thus preventing mental patients from seeking medical treatment [27].

Research in Hong Kong suggest that opposition to mental health facilities is associated with NIMBY sentiments. Opponents believe that facilities should be placed anywhere except their ‘backyard’ [28]. This situation is very similar to those in Canada, the US, Taiwan, and Macau. Residents in these areas share the concerns that the establishment of these facilities in their communities would threaten the safety of personal property and have a negative impact on the reputation of the community [29].

A lack of collaboration between government bodies is another barrier to establishing mental health facilities [30]. Challenges include a lack of Planning Department and Housing Department involvement in welfare planning, a lack of awareness of available land on the part of the Social Welfare Department (SWD), conflicting policies among government departments, and the SWD’s lack of power in interdepartmental negotiation compared to other departments [4]. These challenges are compounded by the current decentralized manner of processing land supplies and reviewing sites for welfare facilities [31].

### Strategies for addressing discrimination

Countries such as New Zealand, Australia, and Canada emphasize social integration through legal provisions to prevent discrimination against people with disabilities. In Japan and Korea, national and local policies for protecting the rights of people with mental illness are less developed. Hong Kong has relatively less-established policies to promote the rights of persons with disabilities and mental illness.

In Australia, the government has launched anti-discrimination regulations to protect people with mental illness [32]. A Hong Kong-based expert on Australian mental health services pointed out that it is illegal to use uncivil language to oppose the establishment of such facilities. Similarly, Canada prohibits prejudice against people with mental disabilities and proposes community residence rights for mental health patients [33, 34]. The US government notes that individuals have the right to be close to mental health facilities in the community [35].

Singapore has attached great importance to mental health treatment and rehabilitation in recent years, and mental health services are integrated into master plans and other regulations to respond to increasing demands for mental health services [36]. Similarly, Taiwan’s People with Disabilities Rights Protection Act protects the equal rights of persons with disabilities [37].

### Official protocols and processes

New Zealand, Australia, and Canada have clear protocols for land zoning for social and mental health services. In contrast, Japan and Korea have adopted a more ‘laissez-faire’ approach, with no formal official policies and protocols for siting community mental health facilities. Similarly, in Hong Kong, land development plans have neglected the assurance of welfare facilities for new communities.

In New Zealand, city councils reserve venues for special uses such as affordable housing and community services. The interests and well-being of the service recipients are of utmost priority [38]. In Canada, provincial and territorial processes adhere to federal legislation to ensure the safety of communities and the success of local projects [39]. Since mental health facilities and other clinics must be situated in sites already zoned for community and welfare purposes, opinions of residents do not affect implementation. In the US, explicit legislation outlining land zoning approaches for different purposes (including mental health services) are generally stricter than those in countries adopting right-based approaches. Singapore also has a sophisticated land zoning system for planning community facilities, including mental health services, and incorporates mental health service development into national planning documents [40, 41].

In Taiwan, there are no specific guidelines for establishing rehabilitation centres in the community. The Handbook for Dealing with Protest against Residence and Community Services for Persons with Disabilities was developed by a patients’ rights group to guide organizations and patients to negotiate with neighbourhood residents and to promote equality in the community [42].

### Consultation processes

In Hong Kong, there is no standardised protocol for public consultation on the establishment of facilities [4]. Even when there is public consultation, some groups such as rehabilitation groups are often overlooked [43]. In Taiwan and Macau, relevant departments rely on community organisations to communicate with residents, leading to a perception that residents are not involved in the decision-making process [4, 44].

In New Zealand and Australia, the governments have clear guidelines for the establishment of public facilities, with an emphasis on transparency and public participation. Information on projects can be accessed on government websites [45]. In Canada, provincial or territorial governments are responsible for the mental health facility siting. Public hearings should be held before project implementation to ensure that residents’ voices will be considered when adjusting plans [39].

Singapore adopts a comprehensive approach to public consultation, which is, however, mainly conducted for macro-level national strategies (e.g. general master zoning plans) or major planning decisions (e.g. amendments to master plans). In Macau, the government arranges public consultations on government projects. One example is the planning processes for rehabilitation services for 2016–2028 [46]. In Taiwan, when privately run community mental health services are established, the government plays a minimal role in promoting the establishment. There are no official public consultation mechanisms and protocols [47]. In Korea, the Central Mental Health Evaluation Committee and Supporting Committee are responsible for assessing demands for mental health care services, but no effective public engagement or consultation approaches have been developed [48]. Japan has no official and institutionalized framework for consulting neighbourhood residents on the establishment of mental health centres [27].

### Strategies for addressing concerns and opposition

New Zealand, Australia, and Canada emphasize community education to increase public awareness of mental illness and reduce stigma and discrimination. Residents are mobilized to participate in relevant policy decision activities. However, in the five Asian jurisdictions, the educational activities for social integration are generally not as structured as those undertaken in the Western countries. In Hong Kong, community education measures and publicity on receptiveness toward mental illness are relatively weak. Community leaders play important roles in the decision-making process concerning service establishment. As residents generally trust these local representatives, it would be difficult to establish community service facilities if these representatives disagree with the establishment [4, 6].

New Zealand uses public education, such as mass media campaigns and local events, to eliminate discrimination and fear toward patients with mental health issues [49]. Similarly, Canada emphasises the importance of publicity, public participation, and public understanding [50]. Provincial and municipal authorities have developed methods of collecting public opinion to address NIMBY sentiment and public opposition [39, 51]. In the US, research recommends engaging local service providers in reforming public perceptions of mental illness and using popular media to disseminate information on mental illness and care [52].

In Singapore, government agencies, healthcare providers, and community partners work closely to reduce social stigma about mental health [53]. In Macau, community education on mental illness has improved in recent years. The government organizes annual activities on Mental Health Day to educate the public about mental illness and reduce discrimination [54, 55]. In Korea, research highlights the importance of positive community awareness and attitudes toward mental illness before developing mental health projects [56]. Community surveys might be conducted to gauge public attitudes before establishing community mental health centres [57].

The Japanese government has made recent efforts to enhance community mental health care and alter public attitudes toward mental illness, including a ‘from institution-based care to community-based care’ reform intended to change public attitudes toward mental health and reorganize psychiatric mental services and community support systems [27]. However, when it comes to community education and mental health awareness, little evidence is available on resources and support from the various levels of government in Japan.

### Tactics and best practices

In Hong Kong, records of District Council meetings provide ideas about how the establishment of mental health facilities was facilitated. They revealed the need for consultation and education on community care and the nature of mental health services; comprehensive plans to minimize the impact of facilities on the community; and coordination between different government departments.

Through the consultation with experts from Australia, Canada and Taiwan, we learned that different jurisdictions have taken different approaches to setting up community facilities for people with mental illness and those in recovery. In Australia and Canada, large-scale public engagement activities are not preferred due to the concern of triggering discriminations and stigma. Approaches to developing community integrations are commonly used, and they include establishing the mental health facilities on sites and premises that have originally been designated for social and community services.

In Taiwan and Japan, mental health services are not recognized as a unique social service category. Purchasing and leasing private properties for serving and accommodating patients and people in recovery in the community could also avoid controversies triggered by public engagement activities.

## Discussion

The establishment of community-based mental health services in Hong Kong is vulnerable to public pressure and the lack of cooperation among government departments, resulting in significant obstacles to the establishment of facilities. This study highlighted common challenges and barriers in different jurisdictions in establishing community-based mental health facilities. The key challenges include public opposition resulting from mental health stigmatization and discrimination and a lack of knowledge about mental illness. NIMBY sentiments are prevalent across jurisdictions, where opponents might acknowledge the service needs, but they believe that facilities should be placed anywhere except their ‘backyard’. A smaller body of literature discussed challenges beyond opposition from neighbourhood residents, noting the effects of a lack of political support from local leaders and elected officials. Finally, issues with the siting process itself, including a lack of transparency, limited collaboration between government bodies, and the complexity and duration of siting processes impede the establishment of the services.

### Key strategies and approaches learned

In many Asian regions or countries, having a mental illness is still an uncomfortable or stigmatized experience. The Hong Kong Government attaches great importance to the mental health of the public, in terms of not only medical care but also the promotion, prevention, early identification and provision of timely intervention, treatment, and rehabilitation services for those in need [58]. On the legal front, Hong Kong's Mental Health Ordinance (Cap 136) is concerned with the care and treatment of people with mental illness, guardianship, administration of welfare, property, and related matters. This Ordinance protects the rights of people with mental illness to treatment, care, guardianship, and other specific benefits. While the Hong Kong Government is committed to eliminating discrimination against particular groups, there remain stereotypes and prejudices against people with mental illness. In this regard, the Disability Discrimination Ordinance in Hong Kong serves to prevent discrimination, harassment, or stigmatisation against people with mental illness or people in recovery and their carers on the basis of the patient's disability. Even though mental health care is relatively common in Hong Kong and it has a relatively well-established legal framework to ensure the rights of people with mental illness and to protect them from discrimination, there are still various obstacles to the establishment of mental health facilities.

Through reviewing the strategies used in different jurisdictions, we have categorized four general approaches to the siting of mental health facilities: a human rights-based approach, a legal-oriented approach, a negotiated approach, and a laissez-faire approach. Countries adopting a *human rights-based approach* include Australia, New Zealand, and Canada. This approach includes legislation against discrimination against people with mental illness, legal protection of statutory rights of people with mental illness, and public education and consultation strategies to facilitate the establishment of community mental health services. Mann et al. [59] also illustrated that respect for human rights leads to improved mental health and could facilitate clinical improvement at a relatively low cost. Mental-health-related legislation can clarify the rights of patients and impose legal responsibilities on the government. When a mental-health-related case arises, the government should be more decisive in handling it under the legislation [60]. Countries adopting a *legal-oriented approach*, such as Singapore and the US, generally adopt explicit legislation outlining land zoning approaches for different community purposes. These include zoning legislation for mental health services, which are stricter than that in countries adopting the human rights-based approach, and legally binding strategies with the goal of establishing social welfare facilities. These legal mechanisms could shorten the time required for establishing social welfare units in communities and neighbourhoods.

Jurisdictions adopting a *negotiated approach*, such as Macao and Taiwan, focus on negotiation and collaboration with community stakeholders when establishing mental health facilities and other sensitive community facilities. Facilities are mainly housed in private properties, and service providers and patients’ groups generally have to negotiate with landlords and local residents or community organizations. Some non-governmental organizations and patients’ groups have developed public engagement strategies based on their past experiences.

Finally, Japan and Korea have adopted a *‘laissez-faire’ approach*, as they do not have formal official and NGO policies and protocols for siting community mental health facilities. Although there are occasional public engagement or consultation activities concerning mental health facilities in some municipalities, these are generally ad hoc activities initiated by local authorities, and facilities are often located in private properties where no extensive public consultation is required. National and local policies for protecting the rights of people with mental illness and disabilities are less developed.

### Relevance to the situation in Hong Kong

When compared with countries adopting the human rights-based approach, Hong Kong has relatively less-established policies promoting the rights of persons with mental illness. The enforcement of existing rights-based policies or legislation is often difficult and time-consuming. Thus, legislation and official guidelines in Hong Kong may not be as effective in protecting the rights of persons with mental illness as those in jurisdictions adopting the human rights-based approach. In contrast to jurisdictions following the legal-oriented approach, land development plans in Hong Kong have long neglected the assurance of welfare facilities that new communities might need. Yet, Hong Kong’s judicial system does have a legal basis to promote human rights practices and protect the rights of people with mental illness. However, it may not be possible to fully imitate the systems and practices in other jurisdictions in the establishment of mental health and other sensitive facilities, since Hong Kong’s town planning and mental health care systems are very different from those of Singapore and Western countries that do not face the same oppositions in local neighbourhoods.

Although discrimination against people with mental illness is common in Japan and Korea, the establishment of community mental health facilities in these two countries is less confrontational when compared to Hong Kong since those facilities are mostly located in private properties and require only the consent of the landlords. These governments basically have no official protocols or strategies to support the service providers in terms of site selection. Therefore, this laissez-faire approach does not resemble the context in Hong Kong.

The situation in Hong Kong is similar to that in Taiwan, where land resources are extremely scarce. With reference to the negotiated approach, establishing social welfare and community service facilities in government buildings or private properties could shorten the time required for facility establishment and reduce disputes with local residents. In Hong Kong, community engagement and consultation are necessary, due to the socio-political expectations of the public. Therefore, a mixed-model approach for consultation and facility establishment could be based on the combined characteristics of the negotiated, human rights-based, and legal-oriented approaches. This mixed-model approach could balance the needs for public involvement using the existing legal framework to protect the vulnerable and minority groups.

### Limitations and future directions of research

A limitation of this study is that the documents and materials related to the policies and protocols in different jurisdictions are scarce. The gaps between documentation and actual implementation practices were unable to be assessed. Regarding the challenges and barriers to the establishment of mental health facilities, the reviewed literature reflects a dominant focus on opposition stemming from neighbourhood residents, with less attention to political and policy barriers. This illustrates the depoliticization of site selection problems, shifting responsibility from policymakers to residents (and their ‘lack of understanding’). Future research should consider not only public opposition and residents’ perspectives, but also wider explicit and hidden political contexts and interests of political stakeholders.

## Conclusion

The public in Hong Kong tends to have a negative perception of people with mental illness. Community education and promotion to raise awareness of needs of people with mental illness in Hong Kong are desperately needed. There is a lack of systematic measures to promote public acceptance of service users and understanding of their service access rights. The enforcement of the existing human right legislation is rare in view of the opposition raised by local residents who also want to claim their civil rights. Consequently, it is challenging to establish facilities providing community mental health support.

Many Western countries generally adopt a more well-established protocol for the establishment of facilities for people with mental illness. They make use of formal legislations and policies to support service provision and protect the rights of people with mental illness. In contrast, while the Hong Kong government has also put in place relevant legislation to protect the rights of people with disabilities, nothing can prevent residents with opposition views to raise excuses such as inadequacy of public consultation or use other reasons such as other more important needs of the local neighbourhood, congested transportation arrangements, or a lack of parking facilities.

To avoid public consultation being used as a stalling strategy by opposition stakeholders, more robust official guidelines on land use planning, legislation, and enforcement of human rights protection should have been formulated and implemented. The government should set up an inter-departmental working group to collect local residents' views. Regular inter-departmental mental health education programmes could be organised throughout the territory to raise awareness of mental health and reduce discrimination against people in recovery. As enforced in other Western countries, the legal human right framework should be implemented proactively to shape policy and protocol development and protect the service access rights of people with mental illness.

## Data Availability

Supporting data and data analysis materials are available from the corresponding author (Daniel, Lai) upon request.
